# Eosinophilic annular erythema in a patient with hepatitis B-related cirrhosis^؆^

**DOI:** 10.1016/j.abd.2026.501362

**Published:** 2026-05-06

**Authors:** Beatriz F. Vilela, Maria Cristina Fialho, Ana Ferreirinha, Cândida Fernandes

**Affiliations:** Department of Dermatovenereology, Santo António dos Capuchos Hospital, Unidade Local de Saúde de São José, Lisbon, Portugal

Dear Editor,

Eosinophilic Annular Erythema (EAE) is a rare, benign dermatosis characterized by annular or polycyclic erythematous plaques and a dermal eosinophil-rich infiltrate on histopathology. Although initially described in children, it has also been reported in adults, occasionally in association with chronic systemic disorders or malignancy.^1^, ^2^

An 89-year-old man with decompensated cirrhosis secondary to chronic hepatitis B infection developed multiple annular plaques on the neck, upper limbs, and proximal thighs during hospital admission. The lesions were erythematous-violaceous, well-demarcated, with central clearing and mild pruritus (Fig. 1A‒B). The patient recalled a similar self-limiting eruption approximately one year earlier.

**Fig. 1:**
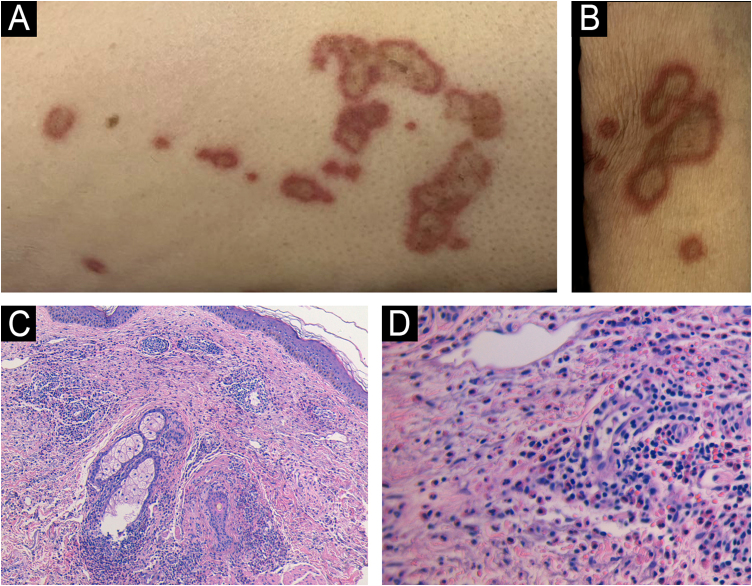
(A) Annular erythematous-violaceous plaques with central clearing on the thigh. (B) Well-demarcated annular plaques on the upper limb. (C) Dense perivascular and interstitial eosinophil-rich dermal infiltrate with edema and erythrocyte extravasation (Hematoxylin & eosin, ×100). (D) Eosinophil-rich infiltrate without vasculitis or flame figures (Hematoxylin & eosin, ×400).

A 4-mm punch biopsy from the thigh revealed a dense perivascular and interstitial dermal infiltrate composed predominantly of eosinophils, with associated dermal edema and erythrocyte extravasation (Fig. 1C‒D). No vasculitis or flame figures were observed. Peripheral blood eosinophil count was within normal limits. The clinico-pathological correlation supported the diagnosis of eosinophilic annular erythema. The lesions gradually resolved over four weeks with only topical corticosteroids.

The differential diagnosis of annular plaques includes tinea corporis, annular urticaria, subacute cutaneous lupus erythematosus, erythema annulare centrifugum, and Wells syndrome.^3^ In EAE, diagnosis relies on clinical morphology together with the characteristic eosinophil-rich dermal infiltrate.

Although often idiopathic, adult-onset EAE has been linked to various systemic conditions, including hepatitis C infection, autoimmune thyroiditis, chronic renal failure, autoimmune pancreatitis, and malignancy.^4^, ^5^ Recent evidence suggests that EAE represents part of a broader spectrum of eosinophilic dermatoses driven by type 2 inflammation, in which IL-5-mediated eosinophil activation and dermal recruitment play a central pathogenic role. This paradigm is supported by reports of therapeutic response to IL-5 blockade in refractory cases.^6^

The association between EAE and liver disease is increasingly recognized. Hepatic dysfunction (whether viral, autoimmune or cholestatic) creates a systemic inflammatory environment characterized by altered cytokine metabolism, impaired antigen clearance, and enhanced Th2-skewed immunity, all of which may facilitate eosinophil activation and dermal migration. Cases linking EAE to autoimmune hepatitis, including situations in which cutaneous lesions preceded the diagnosis of hepatic autoimmunity, reinforce the possibility of EAE acting as a cutaneous marker of evolving liver disease.^7^ Similarly, EAE has also been described in association with primary biliary cholangitis.^8^ In our patient, the onset of EAE during hepatic decompensation suggests that fluctuations in systemic inflammation related to chronic liver disease may act as a triggering factor.

Management of EAE remains challenging due to its relapsing course. Topical corticosteroids are frequently used, but systemic therapy is often required. Antimalarials such as chloroquine and hydroxychloroquine have demonstrated efficacy, although prolonged treatment may be necessary to achieve sustained remission.^9^, ^10^ Other therapeutic options include dapsone, doxycycline, systemic corticosteroids, ciclosporin and methotrexate, with variable responses. In refractory disease, emerging therapies such as JAK inhibitors and biologics targeting Th2 cytokine pathways have shown benefit in isolated cases.^6^, ^8^ This case expands the clinical spectrum of EAE and strengthens the hypothesis that advanced chronic liver disease may contribute to disease expression.

## Authors' contributions

Beatriz F. Vilela: Design and planning of the study; drafting and editing of the manuscript; data survey, collection, analysis and interpretation of data; critical review of the literature; approval of the final version of the manuscript.

Maria Cristina Fialho: Analysis and interpretation of data; intellectual participation in the propaedeutic and/or therapeutic conduct of the studied case; critical review of the literature; approval of the final version of the manuscript.

Ana Ferreirinha: Analysis and interpretation of data; intellectual participation in the propaedeutic and/or therapeutic conduct of the studied case; critical review of the literature; approval of the final version of the manuscript.

Cândida Fernandes: Analysis and interpretation of data; intellectual participation in the propaedeutic and/or therapeutic conduct of the studied case; critical review of the literature; approval of the final version of the manuscript.

## Financial support

None declared.

## Research data availability

Does not apply.

## Conflicts of interest

None declared.

## References

[1] Sempau L., Larralde M., Luna P.C., Casas J., Staiger H. (2012). Eosinophilic annular erythema. Dermatol Online J.

[2] Rongioletti F., Fausti V., Kempf W., Rebora A., Parodi A. (2011). Eosinophilic annular erythema: an expression of the clinical and pathological polymorphism of Wells syndrome. J Am Acad Dermatol.

[3] Gray T., Lee J., Segars K., Knopp E., Miller R. (2021). Eosinophilic annular erythema: a striking clinical presentation with potential systemic implications. JAAD Case Rep.

[4] Heras M.O., Muñoz N.P., Sancho M.I., Millet P.U. (2017). Eosinophilic annular erythema in adults: report of two cases and review of the literature. An Bras Dermatol.

[5] González-López M.A., López-Escobar M., Fernández-Llaca H., González-Vela M.C., López-Brea M. (2015). Eosinophilic annular erythema in a patient with metastatic prostate adenocarcinoma. Int J Dermatol.

[6] Zychowska M., Tutka K., Reich A. (2020). Mepolizumab therapy for recalcitrant eosinophilic annular erythema in an adult: a case report and review of treatment options. Dermatol Ther (Heidelb).

[7] Awosika O., Totoraitis K., Eleryan M., Rengifo-Pardo M., Ehrlich A. (2018). A case of eosinophilic annular erythema as a presenting sign for autoimmune hepatitis. JAAD Case Rep.

[8] Niu Y.-L., He H.-Y., Fang S. (2025). Successful treatment of refractory eosinophilic annular erythema with tofacitinib. An Bras Dermatol.

[9] Ljubojević Hadžavdić S., Bartolić L., Bradamante M. (2018). Prolonged treatment of eosinophilic erythema annulare with chloroquine. Acta Dermatovenerol Croat.

[10] Chastagner M., Shourik J., Jachiet M., Battistella M., Lefevre G., Gibier J.B. (2022). Treatment of eosinophilic annular erythema: retrospective multicenter study and literature review. Ann Dermatol Venereol.

